# Food Insecurity and Blood Pressure in a Multiethnic Population

**DOI:** 10.3390/ijerph20136242

**Published:** 2023-06-28

**Authors:** Claire Townsend Ing, Brettany Clemens, Hyeong Jun Ahn, Joseph Keawe‘aimoku Kaholokula, Peter S. Hovmand, Todd B. Seto, Rachel Novotny

**Affiliations:** 1 Department of Native Hawaiian Health, University of Hawaii at Mānoa, 677 Ala Moana Boulevard, Honolulu, HI 96813, USA; 2 Nancy Atmospera-Walch School of Nursing, University of Hawaii at Mānoa, 2528 McCarthy Mall, Honolulu, HI 96822, USA; 3 Department of Complementary & Integrative Medicine, University of Hawaii at Mānoa, 651 Ilalo Street, Honolulu, HI 96813, USA; 4 Center for Community Health Integration, Case Western Reserve University, 10900 Euclid Avenue, Cleveland, OH 44106, USA; 5 Cardiovascular Diseases, Queen’s Medical Center, 550 S Beretania Street, Honolulu, HI 96813, USA; 6 Human Nutrition, Food and Animal Sciences, University of Hawaii at Mānoa, 1955 East-West Road, Honolulu, HI 96822, USA

**Keywords:** food insecurity, blood pressure, hypertension, Native Hawaiian

## Abstract

Food insecurity is a social determinant of health and is increasingly recognized as a risk factor for hypertension. Native Hawaiians bear a disproportionate burden of hypertension and known risk factors. Despite this, the relative effects of food insecurity and financial instability on blood pressure have yet to be investigated in this population. This study examines the relative effects of food insecurity and financial instability on blood pressure, controlling for potential confounders in a multiethnic sample. Participants (*n* = 124) were recruited from a U.S. Department of Agriculture-funded study called the Children’s Healthy Living Center of Excellence. Biometrics (i.e., blood pressure, weight, and height) were measured. Demographics, physical activity, diet, psychosocial variables, food insecurity, and financial instability were assessed via self-report questionnaires. Hierarchical linear regression models were conducted. Model 1, which included sociodemographic variables and known biological risk factors, explained a small but significant amount of variance in systolic blood pressure. Model 2 added physical activity and daily intake of fruit, fiber, and whole grains, significantly improving the model. Model 3 added financial instability and food insecurity, further improving the model (*R*^2^ = 0.37, *F* = 2.67, *p* = 0.031). Food insecurity, female sex, and BMI were significantly and independently associated with increased systolic blood pressure. These results suggest a direct relationship between food insecurity and systolic blood pressure, which persisted after controlling for physical activity, consumption of fruits, fiber, and whole grains, and BMI. Efforts to reduce food insecurity, particularly among Native Hawaiians, may help reduce hypertension in this high-risk population.

## 1. Introduction

Food insecurity is an underrecognized yet important social determinant of health [1,2,3,4]. Food insecurity is defined as having “inconsistent access to adequate food because of limited financial and other resources” [5]. The prevalence of food insecurity is influenced by the measurement tool and cut-offs applied. According to U.S. Department of Agriculture (USDA) data, approximately 13,070,800 households, or 10.2% of households, in the US experienced low food security at some point in 2021 [6]. The proportion of households experiencing low food security in Hawaii was slightly lower, 9.1%, for the same time period [6]. Other data sources place the prevalence rates much higher [7]. The 2018 Behavioral Risk Factor Surveillance Survey data estimate a 22% prevalence of food insecurity in Hawaii [7].

Native Hawaiians experience a disproportionate burden of food insecurity. National Health Interview Survey data showed that Native Hawaiians and Other Pacific Islanders are four times more likely to be food insecure than Whites [8]. In 2019, more than twice the number of Native Hawaiians received food from a food pantry, church, or shelter in the past 12 months compared to Whites in Hawaii [9]. Native Hawaiians are overrepresented in the Supplemental Nutrition Assistance Program, and Women, Infants, and Children beneficiaries [10,11].

Food insecurity is associated with multiple risk factors for hypertension, such as unhealthy diets, physical inactivity, and overweight/obesity. Studies have indicated that food-insecure participants consume less fruit and vegetables than food-secure participants [12,13,14]. Adults who are food-insecure were less likely to adhere to the recommended physical activity guidelines than those who are food-secure [15]. In addition to an indirect effect through diet and physical activity, food insecurity may also have a direct effect on blood pressure [16]. Findings from the National Longitudinal Study of Adolescent to Adult Health suggest that young adults with food insecurity are more likely to report a hypertension diagnosis compared to young adults who are food secure [17]. Seligman et al. [18] found that food insecurity was associated with a 20% increase in self-reported hypertension among National Health and Nutrition Examination Survey (NHANES) participants. In a review of hypertension guidelines, patients’ financial stability was the most commonly mentioned social hypertension risk factor [19]. The impact of financial stability on health, and vice versa, is increasingly being examined [20,21]. A systematic review found that housing instability and food insecurity are independently associated with cardiovascular disease [22].

Native Hawaiians also suffer disproportionately from hypertension (i.e., systolic blood pressure [SBP] of ≥130 mmHg or diastolic blood pressure [DBP] of ≥80 mmHg) and behavioral/biological risk factors for hypertension (e.g., inadequate physical activity and overweight/obesity). Native Hawaiians have a hypertension prevalence of 50%. Fewer Native Hawaiians meet the recommended physical activity guidelines and report leisure time physical activity than the state as a whole [23,24]. Compared to 55% of non-Hispanic Whites, 75% of Native Hawaiians have overweight/obesity [24]. However, no study to date has examined the relative effects of food insecurity and financial instability on blood pressure among this high-risk population.

To better understand the relationship between food insecurity and blood pressure, we examined the relative effects of food insecurity and financial instability on blood pressure, controlling for sociodemographics, diet, body mass index (BMI), and physical activity in a multiethnic population, including Native Hawaiians. We hypothesized that socioeconomic factors (i.e., food insecurity and financial instability) would account for a greater proportion of variance in blood pressure than sociodemographic factors and health behaviors.

## 2. Materials and Methods

### 2.1. Participants

Participants (*n* = 124) were recruited from a USDA-funded study called the Children’s Healthy Living Center of Excellence (CHL Center). The CHL Center study assessed the effectiveness of an intervention promoting healthy food and physical activity environments in preventing childhood obesity [25]. Data from the CHL Center study included child individual-level data (e.g., diet, anthropometrics, and sociodemographics), household-level data (e.g., household size and composition), and community-level data (e.g., food environment and walkability). The current study augments these data to include parent/caregiver biometrics (i.e., blood pressure, height, and weight), behavioral factors (i.e., diet, physical activity, alcohol use, and tobacco use), sociodemographics (e.g., education, income, and employment), psychosocial factors (i.e., cultural identity and discrimination), behaviors (e.g., alcohol and tobacco use), and household factors (i.e., food insecurity and financial instability). Future publications presenting an analysis of this augmented data set are in process. The analysis presented here includes household size measured by the CHL Center study.

The current study’s eligibility criteria were (1) parent, guardian, or primary caregiver of a CHL Center study participant, (2) residing in the same household as the CHL Center study participant, and (3) ≥ 21 years of age. The exclusion criterion was being pregnant. This study was reviewed and approved by the Institutional Review Board in the Human Studies Program at the University of Hawai’i at Mānoa under the title “Systems Science Informed Multilevel Theoretical Model of Cardiovascular Health in Native Hawaiians,” protocol 2018-01099.

### 2.2. Procedures

In response to the COVID-19 pandemic, data were collected remotely. Participants were mailed a home assessment kit, which included a blood pressure monitor, scale, carpenter’s square, tape measure, and instructions on how to use these to measure blood pressure, weight, and height, respectively. Participants were asked if they preferred to complete hardcopy or online questionnaires. Those who indicated a preference for hardcopy questionnaires received kits that included those forms and an addressed and stamped return envelope. Those who indicated a preference for online questionnaires received a QR code and URL to access their personalized data collection forms. Participants received a $25 gift card to a local drugstore as compensation for their time.

### 2.3. Instruments and Measures

**Demographic variables.** Demographic variables were collected using a demographic form that assessed age, sex, ethnicity, educational attainment, and employment status. Participants provided their age (in years), date of birth, and the date of completion of the demographic form. Participants indicated their sex as either male, female, or other. To determine ethnicity, participants were instructed to select all options that applied from the following categories: Black or African American, White, American Indian or Alaska Native, Asian, and/or Native Hawaiian or Other Pacific Islander. Within the latter three categories, 21 multiple-select response options (e.g., Athabascan, Japanese, and Samoan) and three open-ended response options were provided. To assess educational attainment, participants were asked to select the single answer that best described their highest grade or year of school completed from the following options: never attended school or only attended kindergarten, grades 1 to 8, grades 9 to 11, grade 12 or GED, college or technical school for one to three years, or college for four years or more. To ascertain employment status, participants were asked to select the response(s) that best described their employment status from the following options: employed for wages/salary, self-employed, fishing/farming, student, subsistence, homemaker, out of work for less than one year, out of work for more than one year, retired, and/or unable to work.

**Biometric variables.** Participants measured their SBP and DBP in mmHg with a portable automatic blood pressure machine. Participants were asked to follow a standardized protocol for obtaining blood pressure, which consisted of taking three measures and using the last two measures to obtain the average SBP and DBP. If a participant’s blood pressure exceeded a safe level (i.e., SBP > 180 mmHg or DBP > 120 mmHg), the participant was instructed to wait five minutes and then measure again. If their blood pressure was still elevated, they were asked to see a medical provider as soon as possible. Height and weight were measured with the following procedures and protocols. Weight was measured to the nearest 0.1 kg using portable scales (Taylor Precision Products Taylor Giant Easy to Read Glass Digital Scale). Participants were asked to wear lightweight clothing, take off their shoes, and empty their pockets when taking weight measurements. Height was measured to the nearest 0.1 cm using a tape measure and carpenter’s square. Height and weight were each measured three times. If no two measures among the original three were within two units (e.g., 0.2 kg for weight), three additional measures were made. Participants were also queried for diagnosed hypertension, diagnosed diabetes, and the use of prescribed antihypertensive medications. Participants were asked to list any antihypertensive medications they were taking. Self-reported hypertension has been shown to have a sensitivity of 82–83% when using a reference standard of diagnosed hypertension via chart review [26,27].

**International Physical Activity Questionnaire.** The 27-item International Physical Activity Questionnaire (IPAQ) long-form was used to assess health-related physical activity, including activity related to a respondent’s job (7 items), transportation (6 items), housework (6 items), recreation (6 items) and sedentary time (2 items) [24]. For each subsection (i.e., job, transportation, housework, recreation, and sedentary time), participants were asked, in the last seven days, how many days they engaged in particular activities. Then, participants were asked how many hours and minutes per day they typically spend doing that activity. Additional items were added to each subsection of the IPAQ, asking participants to compare their current level of physical activity with their pre-pandemic level. The IPAQ was scored as the metabolic equivalent of task (MET)-minutes per week using the following formula: MET level × minutes of activity/day × days per week. The IPAQ has been shown to have acceptable measurement properties across diverse samples [28]. Spearman correlation coefficients for test–retest reliability ranged from 0.96 to 0.46, with most around 0.8, and criterion validity for agreement between IPAQ and accelerometers was 0.3 [28].

**Dietary Screener Questionnaire.** Diet was assessed using the National Cancer Institute’s Dietary Screener Questionnaire (DSQ) [29]. This 26-item tool has been used in the Centers for Disease Control and Prevention’s NHANES. Participants were asked, during the past month, how often they consumed various foods and drinks. Then, participants indicated what specific kinds of foods and drinks they consumed within the past month. New questions were added, asking participants to compare their current level of consumption with their pre-pandemic level of consumption of the following food types: fruits, vegetables, sugar, sugar-sweetened beverages, dairy, whole grains, red meat, and processed meat.

The DSQ can provide estimates of dietary intake of fruits and vegetables (cup equivalents), fiber (g), added sugars (tsp), calcium (mg), dairy (cup equivalents), and whole grains (ounce equivalents). The DSQ provides qualitative indicators of the intake frequency of red and processed meat. Convergent validity of the fruit and vegetable items in the DSQ has been examined in relation to serum carotenoids (correlations ranging from 0.11 to 0.55 in women and −0.02 to 0.48 in men) and 24 h food recall (correlation of 0.35 in women and 0.44 in men) [30,31].

**Stress.** Stress was included in this study as a potential confounding variable. It was measured using a single item. Participants were asked about their mental state after the COVID-19 outbreak in Hawaii. The item was, “In general, I feel more stressed”. Participants indicated their agreement on a scale from 1 (strongly agree) to 5 (strongly disagree).

**Financial Instability.** To assess financial instability, participants were asked to consider a hypothetical unexpected $400 expense and to indicate how they would pay for it. The following responses were collapsed and referred to as “cash or its equivalent”: cover it exclusively using cash, savings, or a credit card paid off at the next statement. Examples of other response options were “Put it on a credit card and pay it off over time”, “Use a payday loan, deposit advance, or overdraft”, and “Would not be able to pay for the expense right now”. Those who were able to cover the expense with cash or its equivalent were considered to have greater economic well-being than those who could not.

This item is used in the Federal Reserve’s annual Survey of Household Economics and Decisionmaking (SHED), conducted since 2013 [32]. The survey questions were written by staff at the Federal Reserve Board in collaboration with other Federal Reserve System staff, academics, and professional survey experts [32].

**Food Insecurity.** Food insecurity was assessed by asking participants how often, in the past 12 months, money ran out for food before the end of the month. This item was answered using a five-point Likert scale, ranging from 0 (never) to 4 (always). The food insecurity item was modified from the USDA Core Food Security Module and has been used in the CHL Center study [33,34]. The Core Food Security Module and the USDA Adult Food Security Survey Module have been shown to be valid and reliable among Native Hawaiian and Other Pacific Islander participants [35,36].

### 2.4. Data Reduction and Statistical Analysis

The education variable was collapsed into four levels: 1. Less than high school education, 2. High school education, 3. Some college education, and 4. College graduate or more. Less than high school education included “never attended school or only attended kindergarten” and “grades 1 to 8” (i.e., elementary to middle school). High school education included “grades 9 to 11” (i.e., some high school) and “grade 12 or GED” (i.e., high school graduate). Some college education included “college or technical school for one to three years”. College graduate or more included “college for four years or more”. Marital status was collapsed into three levels: 1. Married, 2. Single, and 3. Interrupted marital status. Single included “single and not living with boyfriend/girlfriend” and “single and living with boyfriend/girlfriend”. Interrupted included divorced, widowed, and separated. Food security was collapsed into binary categories: secure and insecure. Secure included participants who selected never or rarely for the question regarding how often money for food ran out before the end of the month since the COVID-19 outbreak in Hawaii. Food insecurity included participants who selected sometimes, most times, or always for the same question. Respondents were considered to have hypertension if they had been told by a health professional that they had high blood pressure. Participant characteristics were summarized using descriptive statistics. Biometric data were checked to ensure values were within biologically plausible ranges. The distribution of blood pressure and other continuous measures were checked. Data were not found to significantly deviate from the normal distribution using the Kolmogorov–Smirnov test (*p* > 0.05). Frequencies and percentages were calculated for categorical variables. Means and standard deviations were calculated for continuous variables.

Bivariate associations of all pairwise variables were explored by Pearson correlation coefficients for continuous variables and Spearman correlation coefficients for ordinal variables. Multivariable linear regression models were hierarchically constructed with *R*-squared measures, and the *R*-squared measure changes between models were tested by *F* tests. We constructed three models: The first model (Model 1) included age, gender, ethnicity, marital status, education level, and BMI. The second model (Model 2) added physical activity and daily intake of fruit, fiber, and whole grains. The third model (Model 3) added financial instability, food insecurity, and stress. All data analyses were performed in SAS 9.4, and a two-tailed *p*-value of less than 0.05 was considered statistically significant.

## 3. Results

**Descriptive Analysis.** Participants had a mean SBP of 122.63 ± 18.45 and a mean age of 38.83 ± 9.99 years. The majority (59.46%) were female, and 63.09% were Native Hawaiian or Other Pacific Islanders. The mean BMI was 32.24 ± 9.30, which indicates obesity according to guidelines [37]. Approximately two-thirds had low levels of physical activity. Participants consumed an average of 0.89 ± 0.44 cups of fruit, 1.50 ± 0.48 cups of vegetables, and 1.49 ± 0.61 cups of dairy daily, which are below the USDA recommended daily intake [38]. Approximately one-third reported being food insecure (34.92%) and financially unstable (29.37%). Additional descriptive statistics are presented in Table 1.

**Bivariate Analysis.** A bivariate correlation analysis was conducted to examine the association between SBP, food insecurity, financial instability, and potential confounding variables (Table 2). There was a statistically significant positive correlation between SBP and food insecurity (*r* = 0.25, *p* = 0.004). There was a weak, positive correlation between SBP and financial instability (*r* = 0.10, *p* = 0.16); however, this relationship was not statistically significant. Food insecurity and financial instability had a significant positive correlation (*r* = 0.37, *p* < 0.001). Food intake that was significantly associated with SBP included fruit (*r* = −0.21, *p* = 0.01), fiber (*r* = −0.20, *p* = 0.02), and whole grain (*r* = −0.19, *p* = 0.02). BMI was also significantly correlated with SBP (*r* = 0.39, *p* < 0.001). All variables correlated with SBP at *p* < 0.10 were included in the regression models. Stress was also included in the models as a potential confounder.

**Hierarchical Linear Regression Analysis.** Results of the hierarchical linear regression analysis are presented in Table 3. Model 1 included sociodemographic variables (i.e., age, sex, ethnicity, marital status, and education), stress, and BMI. This model explained a significant amount of variance in SBP (*R*^2^ = 0.29, *F* = 4.61, *p* < 0.0001). The female sex, compared to the male sex, was significantly associated with a higher SBP (*B* = 9.89, *SE* = 2.99, *p* = 0.001). BMI was significantly associated with SBP such that as BMI increased, SBP also increased (*B* = 0.80, *SE* = 0.16, *p* < 0.001). Model 2 added physical activity and daily intake of fruit, fiber, and whole grains. The inclusion of these variables significantly improved the model (*R*^2^ = 0.34, *F* = 4.32, *p* = 0.007). Female sex (*B* = 11.58, *SE* = 3.48, *p* = 0.001) and physical activity (*B* = −0.30, *SE* = 0.17, *p* < 0.001) were significantly and independently associated with SBP. Model 3 added financial instability and food insecurity. The inclusion of these variables significantly improved the model (*R*^2^ = 0.37, *F* = 2.67, *p* = 0.031). Female sex (*B* = 10.15, *SE* = 3.52, *p* = 0.005) and BMI (*B* = 0.63, *SE* = 0.17, *p* < 0.001) both remained significantly and independently associated with increased SBP. Food insecurity was also significantly and independently associated with SBP (*B* = 7.09, *SE* = 3.57, *p* = 0.049), such that individuals who experience food insecurity had higher SBP. The Akaike information criterion decreased from Model 1 to Model 2 and then from Model 2 to Model 3 (Model 1 = 1058.30, Model 2 = 1048.65, Model 3 = 1046.37), suggesting that the model fitness improved.

## 4. Discussion

The purpose of this study was to examine the relative effects of food insecurity and financial instability as social determinants of health on blood pressure while controlling for confounding variables, including other social determinants such as education level. We hypothesized that these two social determinants (i.e., food insecurity and financial instability) would account for a greater proportion of variance (Model 3) than sociodemographic factors (Model 1) and health behaviors (Model 2). Our results partially support this hypothesis. We did not find a relationship between financial instability and SBP. However, in the fully adjusted model, food insecurity remained significantly related to SBP. Individuals who experience food insecurity had higher SBP compared to those who do not experience food insecurity.

The previous literature reports an indirect relationship between food insecurity and blood pressure through diet and physical activity [39,40]. A potential explanation for this finding is that, due to financial and time constraints, individuals with food insecurity eat diets high in processed foods and low in fruit, vegetables, and fiber [41,42]. Diets high in processed food and low in fruit, vegetables, and fiber are associated with weight gain and high blood pressure [43,44]. Individuals with food insecurity have higher weight and blood pressure compared to those without food insecurity [15,45]. Additionally, individuals with food insecurity may be less able to participate in an adequate amount of physical activity, further increasing their blood pressure [39,40]. Our data support this relationship. Food insecurity was related to less consumption of fiber, vegetables, and whole grains. Less fiber and whole grain consumption were also related to higher SBP.

Our results also suggest a direct relationship between food insecurity and SBP, which persisted after controlling for physical activity, consumption of fruits, fiber, and whole grains, and BMI. Much of the literature supports a direct relationship between food insecurity and blood pressure [39,46]. A meta-analysis found a positive association between food insecurity and self-reported hypertension [47]. However, this same analysis did not find a significant association between food insecurity and hypertension determined by blood pressure measurement or chart review. A positive association between stress and blood pressure has been found in previous research [48]. Hicken et al. [49] found that vigilance, as a source of chronic stress, contributes to a greater prevalence of hypertension in African American men. In a review of prospective cohort studies, Bergmann et al. [50] found that greater marital stress and greater perceived stress may lead to increased blood pressure.

A possible explanation for this relationship is that the experience of food insecurity is akin to a chronic stressor [51,52]. Multiple studies have found a relationship between food insecurity and stress. Jessiman-Perreault and McIntyre [53] found that food insecurity has a dose-response relationship to poor mental health, and severe food insecurity functions as extreme chronic stress. The prevalence of high levels of stress is linked to increases in food insecurity [54]. Adults with high food insecurity were more likely to report high levels of stress compared to food-secure adults [55]. Leung and Zhou [56] used biological risk as a measure of the body’s physiological response to chronic stress. They found that women, but not men, who experienced food insecurity had increased biological risk, supporting the idea that food insecurity is related to chronic health outcomes via the experience of chronic stress. Other studies, however, did not find a similar relationship. For instance, Trapp et al. [57] found no difference in the levels of perceived stress between low-income mothers with food insecurity and those without.

Our study results should, as always, be interpreted in light of their limitations. The data we present are from a cross-sectional study; thus, causation should not be inferred. Blood pressure measurements were self-reported. While participants received automated blood pressure monitors, multiple-sized cuffs, and detailed instructions, this may be a source of error. Additionally, the data were collected during the first year of the COVID-19 pandemic, from January 2020 to April 2021, which resulted in some data being collected directly and other data remotely. This may have also impacted our sample size, increasing the possibility that the observed associations may be due to chance. Some demographic data were collected in an earlier time period during the CHL Center study, and thus, some characteristics (e.g., education level and income) may have changed during the study period. Nationwide, food insecurity and stress, both psychological and economic, increased among users of food assistance programs during this time [58]. Therefore, the prevalence and/or severity of food insecurity and stress may have increased for this study’s population as well. However, this could also be considered a strength. While the relationships we found may be weaker or impact fewer individuals in non-pandemic times, their presence in this study attests to the vulnerability of our sample population to external shocks. This vulnerability is important to identify and understand from a public health standpoint. Another strength of our study is the composition of our sample population. Our multiethnic sample was composed of parents or caregivers of two- to eight-year-old children residing in six predominantly Native Hawaiian communities on five of the most populated Hawaiian Islands. Thus, the results of this study are broadly generalizable to families of young children in the state.

## 5. Conclusions

This research provides support for the assertion that food insecurity and blood pressure have a significant and independent relationship. Additionally, this relationship persists after accounting for factors with known relationships to blood pressure, including diet, physical activity, BMI, and sociodemographics. Thus, efforts to reduce food insecurity may help reduce hypertension in this high-risk population. Additionally, efforts to reduce hypertension may exert a greater impact if those efforts also examine and address food insecurity.

## Figures and Tables

**Table 1:** Descriptive statistics of the sample population.

Characteristics	N (%) or M ± SD
Systolic blood pressure (mmHg)	122.63 ± 18.45
Age (years)	38.83 ± 9.99
Sex	
Male	59 (39.86%)
Female	88 (59.46%)
Other	1 (0.68%)
Household size	8.04 ± 0.20
Ethnicity	
NHPI	94 (63.09%)
Asian	23 (15.44%)
White	30 (20.13%)
Other	2 (1.34%)
Marital status	
Single	46 (30.87%)
Married	91 (61.07%)
Interrupted marital status	12 (8.05%)
Employment status	
Employed	99 (66.44%)
Unemployed	28 (18.79%)
Retired	4 (2.68%)
Homemaker	17 (11.41%)
Student	1 (0.67%)
Education level	
Less than high school education	3 (2.01%)
High school education	59 (39.60%)
Some college education	39 (26.17%)
College graduate	48 (32.21%)
Stress (1–5)	3.32 ± 1.36
BMI (kg/m²)	32.24 ± 9.30
Physical activity category	
High	43 (28.86%)
Moderate	12 (8.05%)
Low	94 (63.09%)
Sedentary time (min/week)	2127.34 ± 1598.87
Diet components	
Fruit (cups)	0.89 ± 0.44
Vegetables (cups)	1.50 ± 0.38
Fiber (grams)	15.70 ± 3.47
Whole Grains (ounces)	0.73 ± 0.34
Dairy (cups)	1.49 ± 0.61
Added sugar from sweetened beverages (tsp)	9.51 ± 9.42
Food insecurity	44 (34.92%)
Financial instability	37 (29.37%)

Note: NHPI = Native Hawaiian or Other Pacific Islander.

**Table 2:** Bivariate correlations.

	Sex	Age	Edu	Stress	BMI	Smoke	PA	STime	Fruit	Vege	Fiber	WG	Dairy	SSB	FoodIn	FinIn	HHS
SBP	−0.26 *	0.09	−0.19 *	0.10	0.39 *	0.11	0.14	0.05	−0.21 *	−0.06	−0.20 *	−0.19 *	0.08	0.10	0.25 *	0.10	0.09
Sex	-	−0.21 *	0.16	0.19 *	0.06	−0.14	−0.32 *	0.03	0.14	−0.25 *	−0.38 *	−0.08	−0.35 *	−0.29 *	0.01	−0.15	−0.00
Age		-	0.14	−0.15	−0.14	−0.01	−0.03	−0.01	−0.03	0.11	0.18 *	0.07	0.04	−0.13	−0.16	−0.12	0.10
Education level			-	0.11	−0.28 *	−0.35 *	−0.17 *	0.04	0.32 *	0.20 *	0.29 *	0.25 *	0.11	−0.22 *	−0.34 *	0.22 *	−0.16
Stress				-	0.09	0.06	−0.15	0.10	0.00	0.07	0.05	0.00	−0.05	−0.13	0.14	−0.12	−0.08
BMI					-	0.16	0.09	0.04	−0.21 *	−0.18 *	−0.37 *	−0.19 *	0.04	0.16	0.25 *	0.29 *	0.17
Ever smoker						-	−0.06	0.24 *	−0.12	−0.19 *	−0.16	−0.20 *	−0.02	0.22 *	0.41 *	−0.18 *	0.26 *
Physical activity							-	−0.05	−0.06	0.08	0.08	−0.01	0.14	0.11	−0.09	−0.20 *	0.10
Sedentary time (min/week)								-	0.10	−0.13	−0.10	−0.06	0.07	0.23 *	−0.01	−0.09	0.23 *
Fruit (cups)									-	0.42 *	0.39 *	0.42 *	0.33 *	0.33 *	−0.15	−0.21	−0.16
Vegetables (cups)										-	0.63 *	0.36 *	0.19 *	0.06	−0.21 *	−0.38 *	−0.13
Fiber (grams)											-	0.65 *	0.15	−0.31 *	−0.34 *	−0.35 *	−0.23 *
Whole grains (ounces)												-	0.28 *	0.06	−0.29 *	−0.07	−0.16
Dairy (cups)													-	0.37 *	−0.08	0.02	−0.06
Sugar-sweetened beverages (tsp)														-	0.30 *	0.29 *	0.26 *
Food insecurity															-	0.37 *	0.20
Financial instability																-	0.22 *
Household size																	-

Note: SBP = systolic blood pressure, Edu = education level, BMI = body mass index, Smoker = ever smoker, PA = physical activity, STime = sedentary time, Vege = vegetable, WG = whole grain, SSB = sugar from sugar-sweetened beverage, FoodIn = food insecurity, FinIn = financial instability, HHS = household size. * *p* < 0.05.

**Table 3:** Regression analysis of food insecurity and financial stability on systolic blood pressure (*n* = 124).

		Model 1			Model 2			Model 3	
Variable	*B*	*SE*	*p*-Value	*B*	*SE*	*p*-Value	*B*	*SE*	*p*-Value
Sex (male)	9.89	2.99	0.001	11.58	3.48	0.001	10.15	3.52	0.005
Age (yrs)	0.17	0.17	0.33	0.23	0.18	0.19	0.25	0.17	0.14
Ethnicity									
NHPI	−0.95	4.44	0.83	0.56	4.40	0.90	−0.06	4.36	0.99
Asian	4.71	4.90	0.34	5.57	4.84	0.25	4.65	4.80	0.33
Other	20.36	15.89	0.21	26.77	15.69	0.09	26.89	15.50	0.09
Marital Status									
Single	1.74	5.24	0.74	1.95	5.19	0.71	1.07	5.15	0.84
Married	−2.70	4.89	0.58	−1.94	4.83	0.69	−1.91	4.79	0.69
Education Level	−1.73	1.63	0.29	−0.71	1.68	0.67	0.27	1.72	0.87
Stress	1.05	1.08	0.34	1.33	1.07	0.22	0.95	1.11	0.39
BMI	0.80	0.16	<0.001	0.66	0.17	<0.001	0.63	0.17	<0.001
Physical Activity				−0.30	0.17	<0.001	−1.23	2.58	0.64
Foods									
Fruit (cups)				−2.27	3.52	0.52	−4.12	3.61	0.26
Fiber (grams)				−0.57	0.60	0.34	−0.28	0.61	0.45
Whole Grains (ounces)				−5.11	5.28	0.34	−4.05	5.34	0.45
Financial Instability							0.55	3.47	0.88
Food Insecurity							7.09	3.57	0.05
*R* ^2^		0.29			0.34			0.37	
*F* Change					4.32			2.67	
*p*-value					0.007			0.031	

Note: NHPI = Native Hawaiian or Other Pacific Islander; Other Ethnicity = individuals whose self-reported ethnicity did not include NPHI, White, or Asian, *n* = 2.

## Data Availability

The data presented in this study are available upon request from the corresponding author. The data are not publicly available due to privacy and ethical concerns.
